# To: Factors associated with mortality in mechanically ventilated patients with severe acute respiratory syndrome due to COVID-19 evolution

**DOI:** 10.62675/2965-2774.20240192-en

**Published:** 2024-05-27

**Authors:** Abel Arroyo-Sánchez, Rosa Aguirre-Mejía

**Affiliations:** 1 Universidad Privada Antenor Orrego Escuela de Medicina Humana Trujillo Peru Escuela de Medicina Humana, Universidad Privada Antenor Orrego - Trujillo, Peru.; 2 Red Asistencial La Libertad Hospital Víctor Lazarte Echegaray Seguro Social de Salud Trujillo Peru Hospital Víctor Lazarte Echegaray, Red Asistencial La Libertad, Seguro Social de Salud - Trujillo, Peru.

**To the Editor**

Oliveira et al. evaluated the factors associated with mortality in adult patients on mechanical ventilation (MV) with acute respiratory distress syndrome (ARDS) due to coronavirus disease 2019 (COVID-19) in a retrospective multicenter cohort of 425 patients in Brazil.^(1)^ One of the strengths of this study is the selected population, although I would like to make a few comments.

Mechanical ventilation in the prone position is a strongly recommended therapeutic measure, with moderate level of evidence, for patients with moderate/severe ARDS caused by COVID-19.^(2)^ The authors did not use MV as an analysis variable despite including patients with relationship between partial pressure of oxygen and fraction of inspired oxygen (PaO_2_/FiO_2_) < 150mmHg and low respiratory system compliance (Crs) (<30mL/cmH_2_O), intermediate Crs (30mL/cmH_2_O < Crs < 45mL/cmH_2_O), and high Crs (>45mL/cmH_2_O).

The incorporation of MV in the prone position could contribute to the moderate level of existing evidence, in addition to changing the variables reported as significant.

Additionally, Oliveira et al. reported that obesity was associated with increased mortality in adult patients on MV for ARDS due to COVID-19, but this association was not demonstrated in meta-analyses of patients with ARDS without COVID-19,^(3)^ in which obesity had a protective effect (5 studies, n = 1,133 patients; p < 0.01; odds ratio [OR] 0.68; 95% confidence interval [95%CI] 0.57 – 0.80), and of ARDS patients with COVID-19,^(4)^ in which mortality was similar (9 studies, n = 20,597 patients, p = 0.75; OR 0.96, 95%CI 0.74 – 1.25).

On the other hand, Kowsar et al. performed a network meta-analysis of 97 studies with 19,014 patients with COVID-19 (14,359 survivors and 4,655 deaths) and reported that arterial hypertension (p < 0.001), cerebrovascular disease (p < 0.001), and diabetes mellitus (p < 0.001) were the most influential risk factors among nonsurvivors.^(5)^ In addition, the authors did not observe an association between body mass index and mortality.

These preexisting health conditions are strongly related to obesity, and it is likely that the noninclusion of these comorbidities among the variables analyzed by Oliveira et al. could have influenced the statistical association of obesity with mortality in their patients.
