# Supplementary material for: To: Factors associated with mortality in mechanically ventilated patients with severe acute respiratory syndrome due to COVID-19 evolution
Source: Crit Care Sci. 2024 May 27;36:e20240192en. doi: 10.62675/2965-2774.20240192-en (PMC11152438; doi:10.62675/2965-2774.20240192-en)
Supplement: Translation

# Translation: Para: Fatores associados à mortalidade em pacientes ventilados mecanicamente com síndrome respiratória aguda grave por evolução da COVID-19

**DOI:** 10.62675/2965-2774.20240192-pt

**Authors:** Abel Arroyo-Sánchez, Rosa Aguirre-Mejía

**Affiliations:** 1 Trujillo Peru Escuela de Medicina Humana, Universidad Privada Antenor Orrego - Trujillo, Peru.; 2 Trujillo Peru Hospital Víctor Lazarte Echegaray, Red Asistencial La Libertad, Seguro Social de Salud - Trujillo, Peru.

**Ao Editor**

Oliveira et al. avaliaram os fatores associados à mortalidade em 425 pacientes adultos mantidos sob ventilação mecânica (VM) com síndrome do desconforto respiratório agudo (SDRA) devido à doença pelo coronavírus 2019 (COVID-19) em um estudo de coorte retrospectivo e multicêntrico no Brasil.^(1)^ Um dos pontos fortes do estudo é a população selecionada, mas gostaríamos de fazer alguns comentários.

A VM em decúbito ventral é uma medida terapêutica altamente recomendada, com nível moderado de evidência, para pacientes com SRDA moderada/grave causada pela COVID-19.^(2)^ Os autores não utilizaram a VM como variável de análise, apesar de incluírem pacientes com relação entre pressão parcial de oxigênio e fração inspirada de oxigênio (PaO_2_/FiO_2_) < 150mmHg e complacência do sistema respiratório (Crs) baixa (< 30mL/cmH_2_O), intermediária (30mL/cmH_2_O < Crs < 45mL/cmH_2_O) e alta (> 45mL/cmH_2_O).

A incorporação da VM em decúbito ventral poderia contribuir para o nível moderado de evidências existentes, além de alterar as variáveis consideradas significativas.

Além disso, Oliveira et al. relataram que a obesidade estava associada ao aumento de mortalidade em pacientes adultos sob VM com SDRA devido à COVID-19, mas essa associação não foi demonstrada em metanálises de pacientes com SDRA sem COVID-19,^(3)^ cuja obesidade desempenhou efeito protetor (5 estudos; n = 1.133 pacientes; p < 0,01; razão de chances [RC] de 0,68; intervalo de confiança de 95% [IC95%] de 0,57 – 0,80), e de pacientes com SRDA com COVID-19,^(4)^ cuja mortalidade foi semelhante (9 estudos; n = 20.597 pacientes; p = 0,75; RC 0,96; IC95% 0,74 – 1,25).

Por outro lado, Kowsar et al. realizaram uma metanálise em rede de 97 estudos com 19.014 pacientes com COVID-19 (14.359 sobreviventes e 4.655 mortes) e relataram que hipertensão arterial (p < 0,001), doença cerebrovascular (p < 0,001) e *diabetes mellitus* (p < 0,001) foram os fatores de risco mais influentes entre os não sobreviventes.^(5)^ Ademais, os autores não observaram uma associação entre índice de massa corporal e mortalidade.

Essas condições de saúde preexistentes estão fortemente relacionadas à obesidade, e é provável que a não inclusão dessas comorbidades entre as variáveis analisadas por Oliveira et al. possa ter influenciado a associação estatística entre obesidade e mortalidade em seus pacientes.
